# Using a new odour-baited device to explore options for luring and killing outdoor-biting malaria vectors: a report on design and field evaluation of the Mosquito Landing Box

**DOI:** 10.1186/1756-3305-6-137

**Published:** 2013-05-04

**Authors:** Nancy S Matowo, Jason Moore, Salum Mapua, Edith P Madumla, Irene R Moshi, Emanuel W Kaindoa, Stephen P Mwangungulu, Deogratius R Kavishe, Robert D Sumaye, Dickson W Lwetoijera, Fredros O Okumu

**Affiliations:** 1 Environmental Health and Ecological Sciences Thematic Group, Ifakara Health Institute, P.O.Box 53, Ifakara, Tanzania; 2 Faculty of Public Health and Policy, London School of Hygiene and Tropical Medicine, Keppel Street, London, UK; 3 Vector Biology Department, Liverpool School of Hygiene and Tropical Medicine, Liverpool, UK

## Abstract

**Background:**

Mosquitoes that bite people outdoors can sustain malaria transmission even where effective indoor interventions such as bednets or indoor residual spraying are already widely used. Outdoor tools may therefore complement current indoor measures and improve control. We developed and evaluated a prototype mosquito control device, the ‘Mosquito Landing Box’ (MLB), which is baited with human odours and treated with mosquitocidal agents. The findings are used to explore technical options and challenges relevant to luring and killing outdoor-biting malaria vectors in endemic settings.

**Methods:**

Field experiments were conducted in Tanzania to assess if wild host-seeking mosquitoes 1) visited the MLBs, 2) stayed long or left shortly after arrival at the device, 3) visited the devices at times when humans were also outdoors, and 4) could be killed by contaminants applied on the devices. Odours suctioned from volunteer-occupied tents were also evaluated as a potential low-cost bait, by comparing baited and unbaited MLBs.

**Results:**

There were significantly more *Anopheles arabiensis*, *An. funestus*, *Culex* and *Mansonia* mosquitoes visiting baited MLB than unbaited controls (P≤0.028). Increasing sampling frequency from every 120 min to 60 and 30 min led to an increase in vector catches of up to 3.6 fold (P≤0.002), indicating that many mosquitoes visited the device but left shortly afterwards. Outdoor host-seeking activity of malaria vectors peaked between 7:30 and 10:30pm, and between 4:30 and 6:00am, matching durations when locals were also outdoors. Maximum mortality of mosquitoes visiting MLBs sprayed or painted with formulations of candidate mosquitocidal agent (pirimiphos-methyl) was 51%. Odours from volunteer occupied tents attracted significantly more mosquitoes to MLBs than controls (P<0.001).

**Conclusion:**

While odour-baited devices such as the MLBs clearly have potential against outdoor-biting mosquitoes in communities where LLINs are used, candidate contaminants must be those that are effective at ultra-low doses even after short contact periods, since important vector species such as *An. arabiensis* make only brief visits to such devices. Natural human odours suctioned from occupied dwellings could constitute affordable sources of attractants to supplement odour baits for the devices. The killing agents used should be environmentally safe, long lasting, and have different modes of action (other than pyrethroids as used on LLINs), to curb the risk of physiological insecticide resistance.

## Background

Global efforts against malaria have shown great success in recent years. Examples include the scale-up of long-lasting insecticide treated nets (LLINs) and indoor residual spraying (IRS) for the prevention of malaria [1-3] Despite these successes, there remains significant malaria transmission even in communities where coverage with LLINs is already very high [1]. One of the reasons for this residual transmission is that a low but substantial proportion of the transmission now occurs outside dwellings [4,5]. In response to widespread use of LLINs and IRS, mosquito host-seeking behaviour can change opportunistically from indoor-biting during night time to outdoor-biting starting at dusk and continuing beyond dawn [6,7], matching time periods when humans are available outdoors (Moshi *et al.,* unpublished data).

Indoor vector control interventions, LLINs and IRS have greatly reduced densities of *Anopheles gambiae* and *An. funestus*, which was historically the major African malaria vector and was known to bite predominantly indoors [8-10]. However, there are other vector species, which readily bite humans or other vertebrates outdoors [11,12] and are therefore difficult to fully control using only the indoor interventions [4]. These vector behaviours and continuing transmission justify the need for outdoor interventions to complement LLINs and IRS [4].

Blood-seeking mosquitoes identify and find their vertebrate hosts primarily through olfaction [13,14]. Human body emanations, including breath and skin odours, and their components such as lactic acid, ammonia and carbon dioxide (CO_2_) gas are the most dominant attractant cues [13,15]. The attractant compounds or their synthetic equivalents can therefore be exploited to attract host-seeking mosquitoes [16,17]. Over the years, there have been some considerations of mosquito control by way of attracting and killing host-seeking vectors [18-20], most recently exemplified by plans to deploy odour-baited traps against outdoor host-seeking mosquitoes to complement LLINs in western Kenya [21]. Outdoor lure and kill techniques have been successfully evaluated or used against ovipositing mosquitoes [22,23], tsetse flies [24,25] and crop pests [26]. However, other than a few experimental prototypes and expensive commercial traps for small-scale use mainly outside Africa, this technology is not yet available for mass trapping of host-seeking African mosquito populations, even though its success as complementary intervention is highly likely [27].

The aim of this research was to evaluate an experimental prototype of odour-baited mosquito control devices, ‘the Mosquito Landing Box (MLB)’, and to explore technical options and challenges of luring and killing outdoor biting mosquito vectors using such devices in settings where LLINs are already widely used.

## Methods

### Study area

All field experiments were conducted in Lupiro village (8.385°S and 36.670°E) located in Ulanga district, south eastern Tanzania. Lupiro village lies 300 meters above the sea level on the flood plains of the Kilombero River, approximately 26 km south of Ifakara town. Annual rainfall ranges between 1200mm and 1800mm, and annual mean daily temperature between 20°C and 32°C. The majority of houses have clay brick walls, open windows and open eave spaces. The current major malaria vectors in Lupiro are *An. arabiensis* and *An. funestus*. Standard WHO tests recently showed that the *An. arabiensis* here are still 100% susceptible to organochlorines but have slightly reduced susceptibility (92-98%) to common pyrethroids [28], despite widespread use of LLINs in the area since 2008 [9]. Malaria infection rates in this village have been reducing over the past decade [9,29], but new evidence now suggests that the intensity of transmission remains as high as in the pre-intervention years (Kaindoa *et al*., unpublished data).

### Design and construction of odour-baited Mosquito Landing Box (MLB)

**Figure 1:**
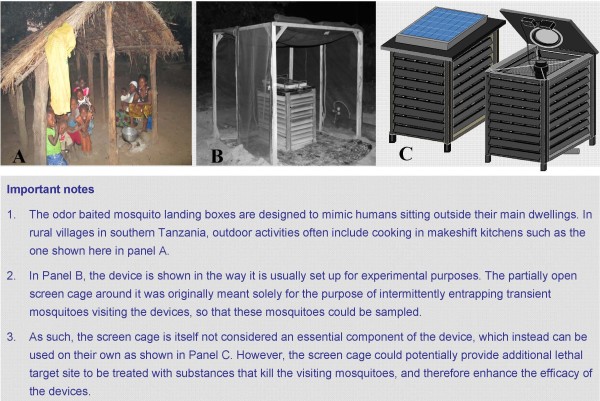
**The odour-baited mosquito landing box.** It is designed to target host-seeking mosquitoes that bite humans outside their houses, e.g. people cooking in open kitchens in rural communities **(A)**. It has a solar panel on its top surface **(C)**, which powers the odour-dispensing system inside it and can be baited with a variety of mosquito attractants including adult human foot odours collected in worn nylon socks, and carbon dioxide gas (as in the experiments described here). A semi-open screen cage (not an essential component of the device) can be used to intermittently entrap and sample host-seeking mosquitoes visiting the device **(B)**. The louvered surfaces are coated with selected mosquito-killing agents, e.g. insecticides diluted in oil paint to withstand outdoor conditions and remain effective for long.

**Figure 2:**
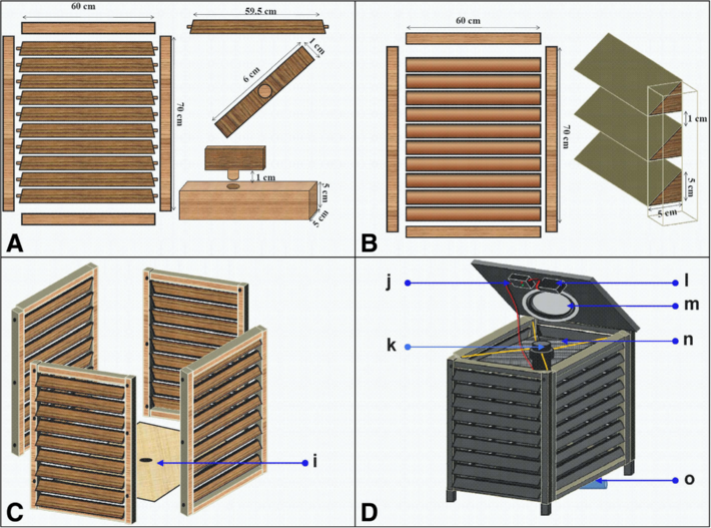
**The design and dimensions of the mosquito landing box, showing side panels with adjustable-angle louvers (A) or fixed-angle louvers (B), which form the mosquito landing surfaces; the side panels are bolted together onto a wooden base (C), and then a top cover and other essential features added (D).** A 20-watt solar panel is securely bolted on top of the device to power the attractant dispensing unit (k). Other features include: i) small aperture through which plastic piping (o) can be inserted to deliver carbon dioxide (CO_2_) gas, j) control unit for the solar energy, k) attractant dispensing unit consisting of a battery-driven 12-volt fan encased in a 5.7cm diameter PVC pipe, l) solar-rechargeable battery m) deflection dish to redirect attractant plumes and n) an inner mosquito contact surface made of carbon netting, that can be treated with mosquito-killing agents.

The MLB (Figure 1A and B) is designed to target disease-transmitting mosquitoes that bite outdoors so as to complement LLINs and IRS. It is a wooden box measuring 0.7 × 0.7 × 0.8m standing on short wooden pedestals raised 10 cm above ground (Figure 2A and B). All sides of the box are detachable, so as to allow easy transport and onsite assemblage. The side panels have multiple louvers (8 or 12) on each of the four sides, which form the mosquito landing surfaces (Figure 2B). The louvers are 1cm wide and are fixed at an angle of approximately 45° facing downwards, ensuring adjustable gaps of at least 2cm between them. The mosquito landing surfaces are covered using substrates that can efficiently deliver killing agents against mosquitoes upon contact. For instance, the surfaces can be covered with oil paints mixed with insecticides [30,31] or with black cotton cloth coated with entomopathogenic fungi, an effective bio-pesticide [32,33].

To improve efficacy of the device, additional mosquito contact surfaces are suspended on the inside of the device (Figure 1C and Figure 2D). These additional contact surfaces, made of UV-resistant shade netting, can also be treated to increase available lethal surfaces, so that mosquitoes that pass through the spaces between the louvers also get contaminated. To address the ongoing problem of physiological insecticide resistance, it may be advisable to use combinations of insecticides of different classes or different modes of action [34].

**Figure 3:**
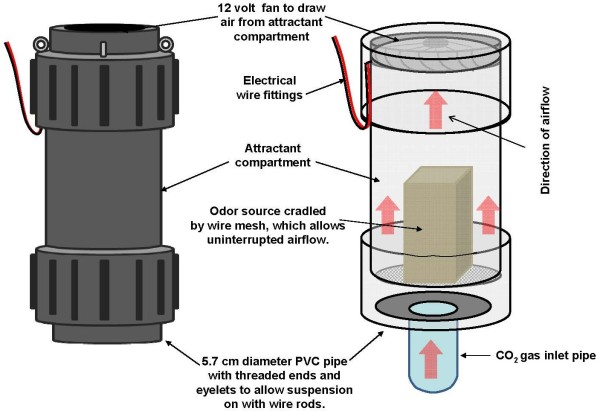
**The odour dispensing unit of the mosquito landing box.** This consists of a PVC pipe (20cm long and 5.7cm diameter), inside which suitable attractants are inserted and the emanating attractants dispersed by air currents generated from a 12-volt fan driven by solar recharged battery. Where necessary, CO_2_ gas can be added into the unit through a plastic pipe fitted from the underside.

The attractant-dispensing unit inside the MLB consists of a short PVC pipe measuring 5.7cm diameter and 20cm length, suspended using expandable wires (Figure 2D). A 12V computer fan is fixed at the top of this PVC pipe. This fan draws air upwards through the attractant compartment, inside which the different baits can be cradled using wire mesh, allowing airflow through the system (Figure 3). The upward air drawn by the fan is redirected by a deflecting dish fitted on the underside of the top cover (Figure 2D), so that the odours come out equally from all four sides of the box. This odour-dispensing system can be fitted with different formats and shapes of odour baits to be inserted into the attractant compartment. A 20W solar panel is securely bolted on top of the device to power the odour-dispenser (Figure 3).

### Mosquito attractants

Within the MLB we used natural human foot odours collected in worn nylon socks and supplemented this with carbon dioxide (CO_2_) gas at 500ml/min to activate mosquitoes and augment attractiveness of the foot odours in the nylon socks [35,36]. Nylon socks are effective methods of collecting, preserving and dispensing foot odours for use in attracting mosquitoes [37]. The nylon socks used here were made of durable but soft microfiber yarns (15 deniers), consisting of 90% polyamide and 10% spandex. The socks were worn by the researchers (NSM and EPM), each time for 10 hours. For each experiment, new freshly worn socks were used and the wearer was not changed until the end of the experiment. The CO_2_ gas was supplied through plastic piping from pressurized gas cylinders via calibrated manual flow meters (Glass Precision Engineering Ltd., United Kingdom). In one of the experiments, host odour suctioned from volunteer occupied tents (instead of real human dwellings) was tested, as a potential low-cost attractant, which would preclude the need for industrial CO_2_.

### Mosquito collection and processing

Since the odour-baited MLB does not have a mosquito-trapping mechanism and is instead designed to only contaminate the attracted mosquitoes, a partially open screen cage made of netting material on a wooden frame (Figure 1B), is used during efficacy evaluation to temporarily entrap transient mosquitoes, which can then be intermittently retrieved at specific intervals during the night. The screen cages are 1.5m × 1.5m and are opened on two adjacent sides when in use, so that the mosquitoes can freely fly towards or away from the MLB, without being substantially interrupted. The two adjacent sides, which are left open to allow mosquito entry, are also fitted with zippers so that they can be intermittently shut to entrap and sample mosquitoes visiting the device. Having the openings on adjacent rather than opposite sides maximizes the possibility of capturing mosquitoes from multiple directions, while minimizing distortions on odour plumes from the MLBs.

Consenting and trained adult volunteers attended to the devices at specified intervals during the night. Each time, they gently closed the two open sides of the screen cages and sampled live mosquitoes using mouth aspirators from: 1) outside surfaces of the MLB itself, 2) the walls, angles and roofing of the screen cage, and 3) the inside surfaces of the MLB where a small number of mosquitoes were occasionally found. Mosquito collection was performed for a standardized period of not less than 5 minutes and not more than 7 minutes, during which the doors of the screen cage remained closed to prevent exit of the entrapped mosquitoes or entry of new mosquitoes during the short sampling period, but also to ensure that any mosquitoes attracted by the volunteer were excluded from possibly being sampled. After each collection, the sides of the screen cage were reopened and the collected mosquitoes kept in labeled cups covered with netting. Collected mosquitoes were killed using petroleum-ether then sorted and identified morphologically into different taxa, and also as unfed, fed, half gravid or fully gravid. A sub-sample of the female *An. gambiae s.l,* and *An. funestus* mosquitoes was preserved in sterile screw-cup micro-centrifuge tubes using dry silica crystals for PCR identification of sibling species [38,39].

### Experimental procedures

#### Tests to determine whether wild free-flying disease-transmitting mosquitoes visit odour-baited MLBs

Two positions were selected in the study village for placing the MLBs, so that the devices were 30 meters apart and at least 10m away from nearest human houses. At first an MLB baited with worn nylon socks and CO_2_ was evaluated against a control, consisting of an un-baited MLB. The two devices were both fitted with the partially open screen cage (Figure 1B), to let in and sample transient host-seeking mosquitoes. Two adult male volunteers were assigned to collect mosquitoes hourly from each position. The baited and the un-baited boxes were rotated nightly to minimize potential bias arising from differences in mosquito densities at the two locations. The volunteers did not rotate, and instead, any differences associated with their skill were considered together with location of the devices as a single source of experimental variation, during the statistical analysis. This experiment was conducted for 22 nights and sampling was carried out hourly, from 6:30pm to 6:30 am each night.

#### Tests to determine whether mosquitoes transiently visited the odour-baited MLBs and left shortly afterwards, and to identify the time of night when these outdoor host-seeking mosquitoes were most active

To develop appropriate mechanisms for targeting mosquitoes visiting the MLBs, it is important to know how long they rest on the surfaces of the devices. While it was not possible to directly measure this period of contact, using our sampling devices, we designed an experiment to simply assess whether mosquitoes that visited the devices actually stayed for long periods around the devices or ‘gave-up’ and left the vicinity of the device shortly after arrival. We used a minimum sampling interval of 30 minutes and maximum of 120 minutes, so that we could make indirect but reasonable inferences simply by correlating the nightly sampling frequencies or sampling intervals, and the total mosquito catches.

We sampled mosquitoes visiting MLBs at different time-intervals: i.e. at increased frequency (sampling every 30 minutes) and at reduced frequency (sampling every 2 hours), relative to the hourly sampling as conducted during the first experiment. Two MLBs baited with worn nylon socks and CO_2_ gas, were assigned specific locations with specific volunteers to sample visiting mosquitoes. The time intervals (30 min, 1 hr or 2 hrs) were assigned randomly so that on any night, a given MLB would be visited by the assigned volunteer either half-hourly, one-hourly or two-hourly. Sampling was conducted from evening to early morning for 21 nights, so that at the end of the experiment, each MLB had been sampled half-hourly for 7 nights, hourly for 7 nights and two-hourly also for 7 nights. This data was also used to estimate the time of night when mosquitoes were most actively seeking hosts outdoors.

#### Tests to determine whether the time of the night when host-seeking mosquitoes are most active around the MLBs matches the time when local people were also active outdoors, and whether these devices could target the same vector sub-populations that would otherwise bite humans outdoors

To ascertain if devices such as the MLB would actually target the specific vector populations that are likely to be transmitting disease to people outdoors, particularly those mosquitoes that bite people outdoors in the early hours of the night or at dawn, it was necessary to directly assess whether malaria vectors are most active around these devices at times of night when people are also performing the common outdoor activities. A series of repeat observations was conducted in 30 randomly selected households in the study village to catalogue the activities that different household members were involved in at different times of the night, beginning at 6:30pm to 6:30am. One member of each candidate household was provided with a printed booklet showing a list of common outdoor activities, so that he/she could record, on a half-hourly basis, activities by any member of the household. These observations were repeated on three different occasions, and the data aggregated by activity and time of night. A full description of this observation method has been described in more detail elsewhere (Moshi *et al*., unpublished).

The half-hourly mosquito collections from the experiment above were also aggregated and host-seeking pattern directly compared to the human outdoor activity pattern from the direct observations.

#### Tests to assess whether presence of the partially open screen cage could influence the number of mosquitoes visiting the device

Two odour-baited MLBs positioned 30 m apart and at least 10 m away from the nearest human houses were used. A volunteer was assigned to each of the MLBs to sample visiting mosquitoes. Over one of the MLBs, the partially open screen cage was fitted and used throughout the night, as described in the previous experiments. On the second MLB however, no such screen cage was fitted, and instead a portable screen cage with all sides blocked was intermittently dropped over the MLB each time the volunteer went to collect the mosquitoes, to entrap mosquitoes that might have been landing on the device or those that were flying around the device at that specific time. Sampling was carried out half-hourly from dusk (6:30pm) to dawn (6:30am) using a mouth aspirator. In both cases, the MLB was opened and gently disturbed at the time of sampling so that any mosquitoes that would be hiding on the surfaces could be detected and sampled. These two set ups were rotated nightly for 12 consecutive days to minimize any bias due to differences between position and volunteers.

#### Tests to determine whether mosquitoes visiting the odour-baited MLB could be contaminated and killed and to identify the main challenges associated with using such devices to kill blood-seeking mosquitoes

Three odour-baited MLBs were assigned specific locations so that they were 30m to 50m apart and 10m to 20m away from the nearest houses. A 50% emulsifiable concentrate of pirimiphos methyl (Syngenta, Switzerland), a mosquitocidal organophosphate recommended for use in IRS, was selected as a candidate mosquito killing agent and used to test this concept. This insecticide was previously reported as effective against indoor biting and as having minimal repellent effects on mosquitoes [40,41]. Also, based on results of a recent study on insecticide bio-efficacy and persistence [28], and on evaluations of lethal odour-baited stations [19], where contact with insecticide was assured, pirimiphos methyl was highly efficacious against local *An. arabiensis* mosquitoes, which are known to be susceptible to organophosphates and organochlorines such as DDT, but have slightly reduced susceptibility to common pyrethroids [28], coupled with substantial behavioral resilience against indoor insecticidal interventions [41]. Treatments were carried out by spraying or painting MLBs using formulations of pirimiphos methyl as follows:

In the first test, two MLBs were sprayed with 1% aqueous solution of pirimiphos methyl, ensuring that all the louvered surfaces were covered, while a third MLB was left unsprayed to act as a control. The three MLBs were covered with the partially open screen cage to sample visiting mosquitoes. However, one of the screen cages covering one of the treated MLBs was also treated (by fitting a rectangular piece of black cotton cloth (150cm × 50cm), soaked in aqueous 1% solution of pirimiphos methyl), to assess whether the screen cage itself would provide additional lethal surfaces to kill the visiting mosquito vectors. The tested devices in this experiment thus included: 1) a control MLB covered with an untreated screen cage, 2) a treated MLB covered with an untreated screen cage, and 3) a treated MLB covered with a treated screen cage. To prevent ants from scavenging upon knocked-down or dead mosquitoes, the set ups were installed on wooden platforms suspended just above ground in bowls of water. Sampling of mosquitoes was carried out half-hourly, and all live and knocked-down mosquitoes collected were maintained on 10% glucose solution inside a holding room at the study site, where their survival was monitored and mortality recorded after 24 hours. Average temperature in the holding room was 30.1°C ± 3.5 by day and 29.0°C ± 2.2 by night, while humidity was 76.4.1% ± 6.6 by day and 81.5 % ± 7.4 by night.

This experiment was first conducted using 1% pirimiphos methyl, but was repeated using 5% formulation in an attempt to guarantee mortality of mosquitoes making only short contact with the device. To test whether it would be possible to increase contact rates of mosquitoes with treated surfaces and minimize decay of candidate killing agents when exposed to natural environmental factors such as rain and sunlight, and to enhance the longevity of treatments applied to the device surfaces, a separate experiment was conducted where an intact MLB (having 12 louvers/side, as described above), was tested alongside another MLB with fewer louvers (8/side), so that there was greater space available for mosquitoes to fly onto the inside treated surfaces (hidden from direct sunlight and rain). These experiments were conducted by painting the MLBs with an oil-based insecticidal paint mixture rather than water-based formulations. Prior to the actual experiments, we performed WHO cone bioassays on treated wood panels to evaluate residual efficacy of the locally prepared formulations of pirimiphos methyl mixed with paint against wild caught malaria vectors. The actual evaluation was then conducted using two MLBs (one with 12 louvers and the other with 8 louvers), both of which were painted with one layer of oil-based paint formulation containing 5% pirimiphos methyl. The treated MLBs were evaluated against a control (an odour-baited untreated MLB with 12 louvers), to assess number of mosquitoes visiting the devices and percentage mortality in each case.

In the final experiment, we attempted to prime the CO_2_ activated mosquitoes to alight and probe more on these devices, so as to possibly increase the period of lethal contact. To do this, we increased the humidity inside the devices by suspending a piece of damp cotton wool (soaked in bowl of clean water), inside a treated MLB, which was then treated by painting with pirimiphos methyl mixed in oil paint as above. The outer and inner surfaces of the MLB louvers, as well as internal suspended netting were treated with 5% pirimiphos-methyl paint mixture. The treated MLB was evaluated against an untreated control for 12 nights, during which the devices were rotated nightly to minimize positional bias.

#### Tests to demonstrate that natural odours suctioned human-occupied dwellings could be used as low-cost bait for the MLBs

A major challenge associated with the strategy of targeting host-seeking mosquitoes outdoors is the need for sustainable sources of effective host odour cues, particularly CO_2_ gas, which is necessary to activate mosquitoes and synergizes with other host odours [16,35,36]. We hypothesized that this challenge could be addressed easily and cheaply by using natural host odours suctioned from local human dwellings, as a low cost and sustainable means of baiting the MLBs or similar devices. Using a technique originally demonstrated by Constantini *et al.,* and Mboera *et al.,*[15,42], we suctioned natural human odours from volunteer-occupied tents (used here instead of actual human occupied dwellings) and channeled these odours into MLBs located 10 m away, through plastic piping of 2.3 cm diameter.

Two locations were identified at the edge of the study village, approximately 50m from the nearest house and canvas tents set up at the sites for volunteers to sleep inside, so as to represent human occupied dwellings. An MLB was set up 10m away from each of the volunteer occupied tents. A plastic pipe, with 2.3cm diameter, was attached on one end to the MLB and on the other end to the volunteer-occupied tent, to form an uninterrupted conduit for host odours from the tent to the MLB. The end of the plastic pipe attached to the tent was fitted with a funnel shaped receptacle (made from locally sourced plumbing hardware) onto which a battery driven 12-volt computer fan was attached to suction out the odours from the tent and into the plastic piping. The other end of the piping was inserted into the MLB such that it was as close as possible to the odour-dispensing unit inside the MLB.

Each night, a comparative test was conducted in which one of the MLBs was supplied with the human odours from volunteer occupied tents (i.e. the odour pipe fitted between the MLB and the tent), while the second MLB was used as control and not supplied with any host odours (i.e. no odour pipe fitted between the MLB and the tent), even though the respective volunteer still slept in the tents nearby. The volunteers in the tents did not rotate between tents, and neither did the individual MLBs. The odour piping was, however, rotated nightly so that at the end of a 12 day rotation, each of the two MLBs had been baited 6 times and un-baited also 6 times. To vary the human odour sources, the same pair of volunteers slept in the tents for only the first 6 nights, and was replaced by a second pair of volunteers for the other 6 nights. Mosquitoes were sampled using screen cages as described earlier and the number of mosquitoes of different taxa caught was compared between baited and un-baited MLBs.

### Data analysis

Statistical analysis was performed using SPSS version 20 (SPSS Inc. Chicago, USA). General linear models (GLM) were fitted and multivariate analysis performed to assess differences between baited MLB and unbaited MLB on catches of different mosquito species. In experiments, to compare effects of different factors, e.g. frequency of sampling on mosquito catches, generalized linear models were fitted using negative binomial distribution with log-link function, and relative rates (and 95% confidence intervals) calculated to estimate mean mosquito catches, relative to the controls. Mosquitoes of different taxa caught were treated as the dependent variable, and modelled as a function of position and treatment as per respective experiments, ensuring that each mosquito species was examined separately. Mean numbers of mosquitoes sampled per night were compared between treatments in the different experiments. In tests of candidate contaminants, percentage mortality of female mosquitoes of different taxa was compared between treatment and controls.

### Protection of research participants

Before embarking on the study, volunteers were provided with explanations of aims and potential benefits and risks, after which written informed consents were obtained from them. To minimize likelihood of any harmful exposure, the volunteers were provided with commercially available mosquito repellent products (consisting of 15% *N, N* diethyl toluamide (Deet)) to protect themselves from bites. A large screened tented area, to provide a protective resting area for use by the volunteers during the experimental period, and long sleeved clothing with ventilated hoods and gloves to prevent bites during mosquito collection were also provided to enhance protection to the volunteers. All participants also had free access to malaria diagnosis by light microscopy and treatment using the first line drug, artemether lumefantrin (Coartem®), if they became unwell, though no volunteer actually became unwell during these experiments. Ethical review and approval was provided by the institutional review board of Ifakara Health Institute (Ref: IHI/IRB/NO.030) and The Medical Research Coordinating Committee at the National Institute of Medical Research in Tanzania (Ref: NIMR/HQ/R.8a/Vol.IX/1222).

## Results

### Tests to determine whether wild free-flying disease-transmitting mosquitoes visit the odour-baited mosquito landing box

**Figure 4:**
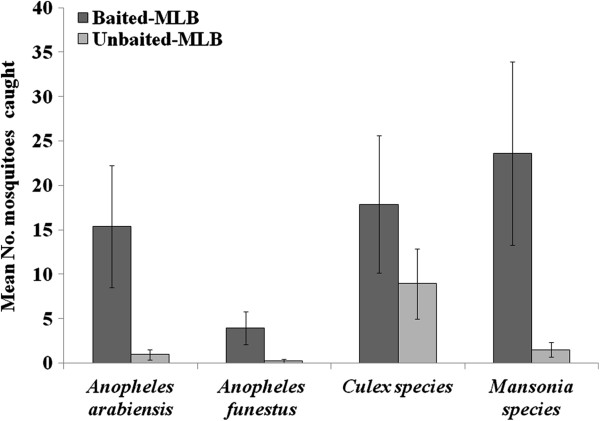
**Model estimated mean number of mosquitoes of different species that visited the odour-baited Mosquito Landing Box (MLB), or the control (unbaited MLB) per night.** The mosquito landing boxes were baited with worn nylon socks and carbon dioxide gas. The Y-error bars represent upper and lower limits of 95% confidence intervals.

As shown in Figure 4, significantly more malaria vectors visiting the baited MLB compared to those visiting the unbaited control (*An. arabiensis* (F = 18.192, df = 1, P < 0.001) and *An. funestus* (F = 21.886, df = 1, P < 0.001)). There were also significantly more *Culex* mosquitoes (F = 12.380, df = 1, P = 0.001), and *Mansonia* mosquitoes (F = 16.264, df = 1, P < 0.001) visiting the treated devices. Location of the devices did not affect numbers of mosquitoes of any species caught visiting the devices (P > 0.05). At least 98% of mosquitoes of each species were unfed, and were therefore likely to be host-seeking.

### Tests to determine whether mosquitoes transiently visited the odour-baited MLBs and left shortly afterwards, and to identify the time of night when these outdoor host-seeking mosquitoes were most active

**Figure 5:**
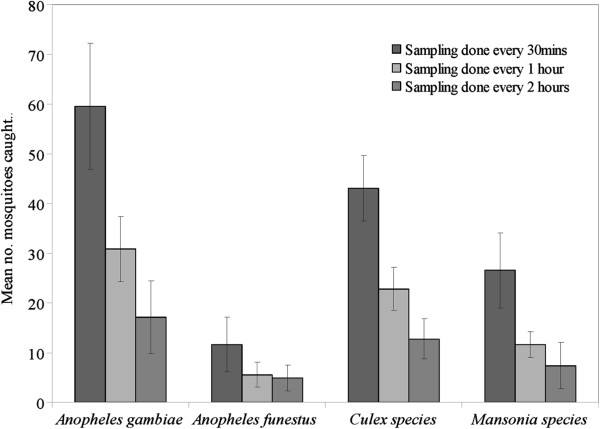
**Model estimated mean number of mosquitoes of different taxa caught visiting the mosquito landing boxes per night, when sampling was conducted at different time intervals (i.e. every 30 minutes, every 1 hour or every 2 hours) each night.** The mosquito landing boxes were baited with worn nylon socks and carbon dioxide gas. The Y-error bars represent upper and lower limits of 95% confidence intervals.

Increasing the frequency of sampling was generally associated with an increase in number of mosquitoes caught visiting the odour-baited MLB (Figure 5), such that the more frequently we sampled mosquitoes on any given night, the more mosquitoes we caught. Relative to 2-hourly sampling, sampling done every 30 minutes yielded 3 times more *An. arabiensis* (RR = 3.254 (1.523 -6.955), df =1, P = 0.002), 2.5 times more *An. funestus* (RR = 2.420 (1.080 - 5.413), df =1, P = 0.32), 3.6 times more *Culex* mosquitoes (RR = 3.553 (1.657 - 7.619), df = 1, P=0.001) and also 3.6 times more *Mansonia* mosquitoes (RR = 3.621 (1.666 - 7.870), df = 1, P = 0.001). Similarly, relative to hourly sampling, we observed that increasing the frequency to half-hourly sampling yielded 1.5 times more *An. arabiensis* (RR = 1.664 (0.766 - 3.614), df = 1, P = 0.198), slightly higher catches of *An. funestus* (RR = 1.156 (0.506 - 2.638), df = 1, P = 0.731), nearly 2 times more *Culex* mosquitoes (RR = 1.831 (0.835 - 4.014), df = 1, P = 0.131), and 1.5 times more *Mansonia* mosquitoes (RR = 1.544 (0.695 - 3.427), df = 1, P = 0.286). The total number of mosquitoes caught with one-hourly sampling was also greater than the total number when doing 2-hourly catches (Figure 5), even though in this comparision, our statistical analysis revealed no significant difference for *An. arabiensis* (RR = 0.601 (0.277 - 1.305), df = 1, P = 0.198), *An. funestus* (RR = 0.865 (0.379 - 1.975), df = 1, P = 0.731), *Culex* mosquitoes (RR = 0.546 (0.249 - 1.197), df = 1, P = 0.131) or *Mansonia* mosquitoes (RR = 0.648 (0.292 - 1.438), df = 1, P = 0.286).

**Figure 6:**
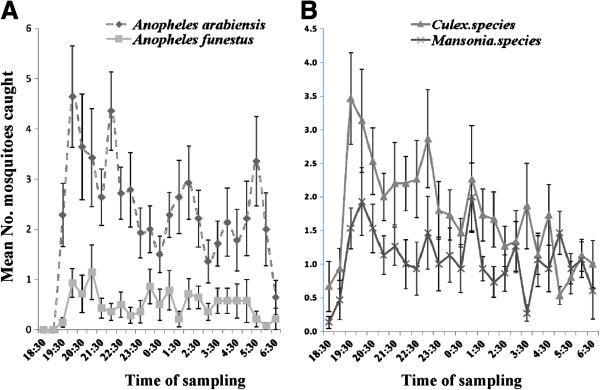
**Host-seeking activity of malaria vectors, *Anopheles arabiensis *and *Anopheles funestus *(Panel A) and Culicines (Panel B) at different times of the night as observed based on half hourly sampling.** The peak visiting time for the malaria vector, *Anopheles arabiensis* occurred in the early hours of the night, i.e. between 7:30 pm and 10:30 pm, followed by a smaller peak around midnight, i.e. between 0:30 am and 2:30 am and in the morning hours, i.e. between 4:30 am and 06:00 am (**A**). For *An. funestus,* the second and third peaks were not obvious. Trends for *Culex* and *Mansonia* mosquitoes showed the first host-seeking peak at the start of the night, but there were no obvious peaks around midnight or dawn, as in the case of *An. arabiensis*.

The peak visiting time for *An. arabiensis* occurred in the early hours of the night, i.e. between 7:30pm and 10:30pm, followed by smaller peaks around midnight, i.e. 0:30 am to 2:30 am and in the morning hours between 4:0 am and 6:00 am (Figure 6). For *An. funestus* mosquitoes however, the second and third peaks were non-existent. Trends for *Culex* and *Mansonia* also showed peaks at the start of the night, but there were no obvious peaks around midnight or dawn, as in the case of *An. arabiensis* (Figure 6). At least 98% of caught mosquitoes were unfed, thus likely to be host-seeking.

### Tests to verify that the visiting mosquitoes actually contacted the surfaces of the MLB, and to assess whether presence of the screen cage influenced efficacy of the devices

The presence of the screen cage around the device did not influence catches of mosquitoes of any taxa (Table 1), and mosquitoes were found on and around the MLB even in the absence of the screen cage, suggesting high likelihood of contact with the MLB, since there was no other alternative landing surface at the sites, other than the MLB itself. There was no statistically significant difference in the number of mosquitoes caught visiting the MLB that was only intermittedly covered with the closed screen cage compared to the catches at the MLB continuously covered with semi-open screen cage throughout the night (P>0.05).

**Table 1:** Estimated mean number of mosquitoes sampled per night from around the odour baited mosquito landing boxes, whenever the boxes were screened (having a semi-open screen cage permanently located over the device throughout the sampling night) and un-screened (the screen cage being used only intermittently at times of mosquito sampling)

**Treatments**	***Anopheles arabiensis***		***Anopheles funestus***		***Culex *species**		***Mansonia *species**		**Total No. mosquitoes**
	Mean (95%CI)	Sum (%)	Mean (95%CL)	Sum (%)	Mean (95%CL)	Sum (%)	Mean (95%CL)	Sum (%)	
Screen cage fixed over the device	45.2	542	2.3	27	22.8	273	13.7	164	1006
	(25.5-80.0)	(53.9)	(1.1-4.4)	(2.7)	(12.8-40.6)	(27.1)	(7.6-24.6)	(16.3)	(100%)
Screen cage used intermittently	44.8	538	2.6	31	20.1	241	16.3	195	1005
	(25.3-79.4)	(53.5)	(1.3-5.0)	(3.1)	(11.3-35.9)	(24.0)	(9.1-29.1)	(19.4)	(100%)

### Tests to determine whether the time of the night when host-seeking mosquitoes are most active around the MLBs matches the time when local people were also active outdoors, and whether these devices could target the same vector sub-populations that would otherwise bite humans outdoors

**Figure 7:**
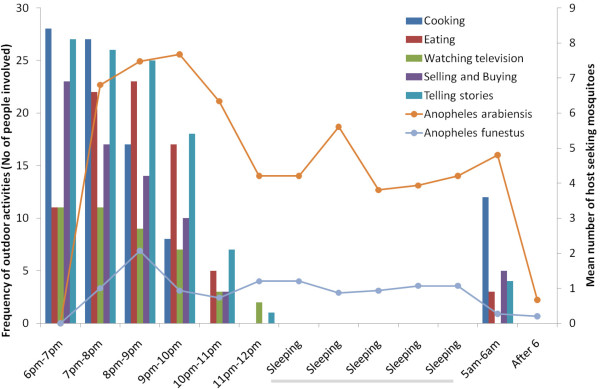
**Correlation between the time when local people are performing various outdoor tasks and the times when host-seeking disease-transmitting mosquitoes are most active outdoors.** Most outdoor activities occur before 11:30 pm and after 5:00 am, which coincides with the time when host-seeking *Anopheles arabiensis* and *Anopheles funestus* (mostly *Anopheles rivulorum*) mosquitoes were most active outdoors.

We observed that the most common outdoor activities were cooking, eating, watching television, telling stories, as well as buying and selling foodstuffs and other commodities (Figure 7). These activities took place mostly before 11:30 pm, and also after 5 am, matching the times when host-seeking *An. arabiensis* and *An. funestus* mosquitoes were also shown to be most active around the MLBs (Figure 6 and Figure 7), suggesting that devices such as the MLB could be used to target the same sub-populations of malaria vectors as those that would otherwise be biting humans outdoors. Reasons associated with these activities being conducted outdoors, and the overall community perception towards outdoor exposure to mosquito-borne pathogens are reported elsewhere (Moshi, *et al.* unpublished data).

### Tests to determine whether mosquitoes visiting the MLB could be contaminated and killed

**Figure 8:**
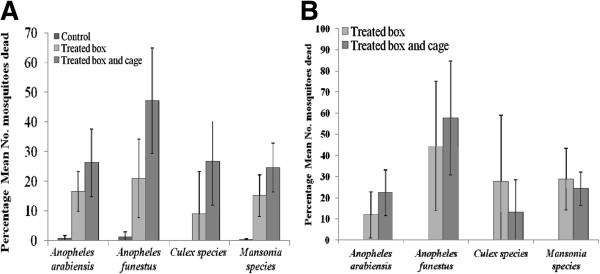
**Mean percentage mortality of mosquitoes, that visited the contaminated odour-baited mosquito landing box (MLB) and controls.** The MLBs were baited with smelly socks and carbon dioxide gas and were contaminated using a water based formulation containing 1% pirimiphos-methyl (**A**) or 5% pirimiphos methyl (**B**). In Panel B, the percentage mortality in the controls was zero, and is therefore not shown. The Y-error bars represent upper and lower limits of 95% confidence intervals.

In the initial test, conducted using water-based formulation of 1% pirimiphos methyl, the mean percentage mortality observed among mosquitoes caught visiting the treated MLB, or the treated MLB covered with a treated cage, was significantly higher than the untreated box (control), for which mortality was either zero or near zero percent (Figure 8). The mortality was however generally low for *An. arabiensis*, being 16% in the set up with treated MLB only and 26% in the set up with treated MLB plus treated screen cage, compared to 1% in the control. Mortality for *An. funestus* was 21% in the set up with treated MLB only, 47% in the set up with treated MLB and treated screen cage and 1% in the control, which suggests that *An. funestus* were more amenable to effects of these treatments than *An. arabiensis*, perhaps because they spent more time on the treated surfaces, as originally shown by Davidson [43].

In tests using aqueous 5% pirimiphos methyl (Figure 8), 24-hour mortality was 58% among the *An. funestus* mosquitoes collected at the treated MLB covered with treated screen cage, and 42% among those collected around the set up having just the treated MLB. Mortality of *An. arabiensis* in this test was, however, only marginally increased, emphasizing the potential resilience of this species against insecticidal intervention outdoors [44].

In tests using oil based mixtures of pirimiphos methyl, more open spaces between louvers on the MLBs and improved humidity inside the devices, we observed that percentage mortality of mosquitoes visiting the devices remained modest over the 4 week test period (Table 2). Maximum 24-hour mortality was 32% for *An. arabiensis* and 33% for *An. funestus* in experiments using paint-based mixture containing 1% pirimiphos methyl. However, in tests using paint mixtures containing 5% pirimiphos methyl percentage 24-hour mortality was 51% for *An. arabiensis* and 25% for *An. funestus* (Table 2).

**Table 2:** Comparison of percentages of mosquitoes (± 2SE) that died after visiting insecticide-treated odour-baited mosquito landing boxes or the controls

		***Anopheles arabiensis***	***Anopheles funestus***	**Other *Anopheles***	***Culex *species**	***Mansonia *species**
Tests conducted using 1% pirimiphos methyl mixed in paint	Control	0.32 ± 0.622	0.89 ± 0.000	0.00 ± 1.441	0.00 ± 0.000	0.00 ± 0.000
	Treated MLB with 12 louvers	6.78 ± 2.769	17.95 ± 8.820	16.23 ± 9.944	2.16 ± 2.584	7.58 ± 4.316
	Treated MLB with 8 louvers and treated internal surfaces	32.07 ± 9.016	33.91 ± 13.200	33.03 ± 12.827	22.20 ± 14.324	25.69 ± 13.429
Tests conducted using 5% pirimiphos methyl mixed in paint	Control	4.07 ± 3.741	0.00 ± 0.000	No data	1.09 ± 1.442	1.62 ± 1.106
	Treated MLB with 8 louvers and treated internal surfaces	50.64 ± 5.128	25.00 ± 13.056	No data	37.76 ± 7.311	47.98 ± 11.315

The MLB was painted with locally prepared pirimiphos-methyl oil based paint while the inner suspended net was also treated by soaking it in the same mixture of pirimiphos-methyl then drying under a shade.

### Tests to demonstrate that natural odours suctioned from human-occupied tents could be used as low-cost bait for the MLBs

The MLB baited with odours channeled from volunteer-occupied tents (used here to demonstrate that natural mosquito attractants can be readily obtained from human dwellings) had significantly more mosquitoes of all species (P<0.05, df=1), visiting it than the unbaited MLB (Table 3). However, we also observed that there were still a substantial number of mosquitoes visiting the baited box, possibly due to interception of these mosquitoes by the devices during flight, or due to residual host odour, since the odour-piping was made of plastic and was rotated nightly.

**Table 3:** Comparison of the estimated mean number of the mosquitoes collected when the odour-baited mosquito landing boxes were baited with host odour suctioned from human occupied tent against un-baited boxes (control)

**Treatments**	***Anopheles arabiensis***		***Anopheles funestus***		***Culex *species**		***Mansonia *species**		**Total No. mosquitoes**
	Mean (95%CL)	Sum (%)	Mean (95%CL)	Sum (%)	Mean (95%CL)	Sum (%)	Mean (95%CL)	Sum (%)	
MLB baited with host odours	18.2	218	1.0	12	38.2	458	37.0	444	1132
	(10.2-32.5)	(19.3)	(0.5-2.2)	(1.1)	(21.5-67.7)	(40.5)	(20.9-65.7)	(39.2)	(100%)
Control (MLB unbaited)	8.0	95	0.2	02	20.3	243	12.6	151	491
	(4.3-14.4)	(19.3)	(0.0-0.7)	(0.4)	(11.3-36.2)	(49.5)	(7.0-22.7)	(30.8)	(100%)

### Sibling species composition and *Plasmodium falciparum* infection rates among the *An. gambiae* complex and *An. funestus* mosquitoes caught during the study

A total of 454 *An. gambiae s.l.* mosquitoes and 717 *An. funestus s.l*, randomly selected from catches in the different experiments were analyzed by PCR [38,39]. Of the successful amplifications, 89% of the *An. funestus* mosquitoes were determined to be *An. rivulorum*, while 11 % were *An. funestus s.s.* However, 100% of all the *An. gambiae s.l*. mosquitoes were determined to be *An. arabiensis*. On the other hand, a total of 567 *An. funestus* s.l and 1539 *An. arabiensis* mosquitoes were tested for *P. falciparum* circumsporozoite protein, using a modified ELISA technique that includes boiling the ELISA lysate for 10 minutes at 100°C to exclude false positives [45]. Of these, 12 individuals from the *An. funestus* group (2.11%) and 7 *An. arabiensis* (0.45%) were confirmed positive.

## Discussion

In addition to ongoing malaria vector control programs, complementary interventions will be necessary to drive the persistent residual transmission towards zero [4,46]. Though malaria transmission in Africa still occurs overwhelmingly indoors [47,48], the proportion that occurs outdoors is steadily increasing, especially after widespread use of indoor insecticidal interventions [6,7], thus the need for tools targeting outdoor transmission [4,5,46]. Odour-baited technologies have been proposed as one of the potential new tools, not only for sampling, but also for controlling mosquitoes outside dwellings [27,49].

While similar technologies have been successfully used against several disease-transmitting arthropods such as tsetse flies (*Glossina* species), horse flies (*Tabanus* species) and stable flies (*Stomoxys* species) [18,24,25,50], applications for mosquito control or malaria control remain absent, and in most cases restricted to small scale experimental set ups. However, recent advances suggest that this technology can have the desired potential to complement existing interventions [16,21,27]. It is therefore important to examine this technology and its potential application in addressing current and future malaria control challenges, particularly those arising from the different biting or resting behaviors of vectors in settings where indoor interventions like LLINs and IRS are already widely used.

The nature of odour plumes arising from the devices and their potential influence on mosquito host-seeking [51,52] was not studied here, so it is not possible to determine whether the MLB actually mimics vertebrate hosts outside their dwellings. Nevertheless, this study demonstrated that such devices, if baited with host-derived odour cues, can attract disease-transmitting mosquitoes including major malaria vectors, *An. arabiensis* and *An. funestus* (most of which were molecularly identified as *An. rivulorum,* a species known to contribute significant transmission in areas where populations of primary vectors have already been drastically reduced by indoor interventions [53-56]). One major challenge previously facing application of odour-baited technologies was the lack of effective lures for large-scale operations but recent developments show that these lures can be constituted readily [16,17], and there is at least one ongoing attempt to use these lures for community–wide malaria control in rural Africa [21]. The MLB is specifically designed to meet the demands of low and middle income countries, so to further improve its acceptability and sustainability, the same solar energy that powers the odour dispensing unit of the devices could also be used for basic lighting in nearby houses, in which case the panel would be positioned on the user’s roof top or on the device, as in these experimental prototypes. This approach would also reduce risks of the MLB being damaged or stolen

Increasing the frequency of sampling was associated with an increase in number of mosquitoes caught visiting the devices, suggesting that mosquitoes spend only brief periods on the devices and that they leave shortly after arrival, presumably on realization that the devices actually offer no real blood meals. Since these experiments were done on non-treated MLBs, the brief visits are not in any way due to potential repellency of any treatments, but instead reflect the natural response by the mosquitoes around outdoor targets. The observations are therefore comparable to those made in experimental huts, where even untreated nets induced early exit of *An. arabiensis*[41]. Evidence suggests that *An. arabiensis*, which now predominates many transmission ecosystems [8,57,58], even if physiologically susceptible, are not readily killed by IRS and LLINs [41,59]. Unfortunately, it is the behavioral resilience rather than physiological insusceptibility, which presents a greater challenge to malaria control than the more commonly assessed physiological resistance [44]. In the current study, the behaviour was exhibited by the very brief visits to the MLBs and the low mortality rates, clearly suggesting that the vectors are equally difficult to control even with outdoor lure and kill stations, unless more innovative approaches are used. While it could be possible to mathematically extrapolate the mean resting times of the mosquitoes on the devices, the aim of this experiment was merely to demonstrate that mosquitoes do not spend long periods of time on these devices, rather than calculate the actual time spent. Our results therefore allow for arguments only on the basis of 30 minutes sampling, being the minimum interval we tested, and it can be concluded that most mosquitoes spent 30 minutes or less on these devices during any single visit. To be able to know exactly how long the insects spend on the devices, the experiment would have to be re-done with multiple frequencies at intervals lower 30mins

To sufficiently contaminate and kill the vectors, agents to be applied onto these devices should be those that act at ultra small doses, or those that can multiply on the mosquitoes once picked up, for example spores of entomopathogenic fungi [20,32]. In an earlier related study (details and data not presented here), we placed two fungus-coated MLBs (baited with worn socks and CO_2_ gas), inside a large screened-cage and released 400 laboratory reared female *An. arabiensis* nightly. Two exposure-free tent traps [60] with sleeping volunteers were also placed in a cage, so that the mosquitoes had a choice between real human odours and the MLBs. Nearly half of mosquitoes recaptured inside the tent traps and a quarter of those recaptured outside the tent traps had fungus growth on their cadavers, confirming that even where there are competing cues from real humans, mosquitoes can still visit the outdoor devices and get contaminated before reaching their target humans (Lwetoijera *et al.,* unpublished). Other than fungal spores, perhaps juvenile insect hormones, such as pyriproxifen, which have a variety of effects on different mosquito life cycle stages and are known to be effective in extremely small doses [61] or even electric grids [62,63] if attached to the device surfaces, might also be used.

The other option for killing the transient mosquitoes that are also resilient would be to carefully use treatments with higher doses of regular insecticides already being used in malaria control for IRS. Assuming that higher doses would lead to greater mortality, properly packaged substrates containing the insecticides, preferably non-pyrethroids (already widely used on LLINs and IRS) could be applied on the MLBs, in a manner that they are protected from environmental effects such as direct sunlight, and would be effective for longer periods. Current vector control technologies that employ increased insecticide dosage to enhance efficacy include PermaNet 3.0® (Vestergard Frandsen, Switzerland), in which the deltamethrin concentration on net surfaces is higher than the concentrations used on the earlier PermaNet 2.0® [64], and durable wall linings which also have higher insecticide doses and lower insecticide decay rates than regular IRS treatments [65]. In the current study, increasing insecticide dose from 1% to 5% was associated with an increase in mortality of the malaria vectors, though the overall effect was still modest (Figure 8). Using paint-based insecticide mixtures and treating the inside landing surfaces of the MLB, would further ensure efficacy under environmental conditions, lower release rates of the active ingredients and reduce decay rates, and it would also improve environmental safety of these devices when treated with chemicals [66]. Gradual release of effective insecticides formulated in paint mixtures has already been achieved, one successful example being the commercial insecticide paint product, Inesfly 5A IGR™, which contains two organophosphates (OPs), chlorpyrifos and diazinon, and an insect growth regulator (IGR), pyriproxyfen [30,31].

Given the risk of insecticide resistance in communities where malaria prevention already involves massive use of insecticidal products, it would be best to prioritize slow-killing non-chemical approaches such as use of entomopathogenic fungus [67], or insecticide combinations, mosaics and mixtures consisting of insecticides of different classes with different modes of action, as proposed by WHO [34]. For example, in places where pyrethroid based LLINs are already being used, it may be preferable that the MLBs are treated with organophosphates or carbamates, so as to avoid the spread of physiological insecticide resistance [34]. Similarly it may be preferable to use chemical insecticides outside the MLB louvers and entomopathogenic fungi on the inside netting surfaces (Figure 1).

The time when mosquitoes were most actively seeking hosts outdoors, as depicted by visits to the MLB, clearly matched the time when local people were also outdoors (i.e. in the early hours of the night and early morning hours), and therefore most exposed to outdoor disease transmission (Figure 6 and Figure 7). While this observation empasizes the need to consider both human and mosquito behaviours in designing new interventions [68], it also reinforces the potential of MLB-like devices to target mosquitoes that would normally bite people outside their homes. Previous studies in Tanzania, have also noted that outdoor activities may be the key drivers of exposure to potentially infectious bites [69], and cause sub-optimal coverage of current indoor interventions such as LLINs and IRS [70]

Despite using evidently sub-optimal formulations of killing agents, the MLBs achieved up to 51% mortality against *An arabiensis*, suggesting that properly formulated mixtures could achieve even greater and longer-term effects. While the maximum efficacy in this study was much higher than 29%, as recently achieved by LLIN/IRS combinations against indoor *An. arabiensis* in the same area [41], this prototype still needs improvement to match the target product profiles earlier described for odour-baited technologies, which assumed 100% mortality of mosquitoes attracted to the devices [27].

An interesting observation from the molecular analysis was that high proportions of mosquitoes in the *An. funestus* group were *An. rivulorum.* This species is known to bite humans predominantly outdoors [71,72] and is often considered of secondary importance because of low vectorial capacity [73], but studies have shown it could be an important vector of *Plasmodium* parasites, especially in areas where LLINs and IRS are already widely used and where overall malaria transmission has been lowered [53-55]. In one of our experiments, higher percentage mortality was observed among the *An. funestus* group than *An. arabiensis* mosquitoes, suggesting these mosquitoes possibly stayed longer on the treated surfaces of the MLB, than *An. arabiensis.* Similar observations were first made in experimental huts in the 1950s by Davidson [43], and they highlight potential differences in performance of lethal outdoor devices against different vector species.

Perhaps the greatest technical challenge facing devices designed to attract and kill disease transmitting mosquitoes on programmatic scales is their overwhelming dependence on CO_2_ gas. Recent advancements such as production of CO_*2*_ from fermentation of sugars using yeast [74,75] can solve this problem to a small extent, particularly for experimental sampling, but their utilization remains costly and labor intensive. Future developments of lure and kill devices must maintain focus on economics of CO_2_. Specific targets should include cheaper sources of CO_2_, lures that do not require augmentation with CO_2_, or new chemicals that have similar effects as CO_2_, for example, 2-butanone, recently tested against anthropophilic vectors [76]. Obviously, one of the easier technical options would be to pipe natural host odours, which consist of exhaled CO_2_, directly from nearby human dwellings, thus minimizing costs associated with maintenance of the programs.

In this study, we have presented results showing that indeed host cues suctioned from human-occupied tents (which we used in this case to represent dwellings), can be efficient baits in the MLB. The actual concept has been investigated widely, particularly in studies of attractiveness of humans to mosquitoes [15,77], in studies aimed at trap development and evaluation against host-seeking vectors [42], and also to study zoophilly versus anthropophilly among malaria vectors [78], yet it has never been considered an option for outdoor mosquito control. In practice, such house-derived host odours could be used alone or supplemented with other cues to improve attractiveness and the overall effectiveness of the technology. In fact, previous studies have shown that adding synthetic mixtures of attractants inside huts in which there were adult volunteers sleeping under bed nets, increased the number mosquitoes entering those huts [16], suggesting that suctioning host odours from nearby households to supplement baits in the MLB would be not only cheaper than using industrial CO_2_ gas, but it would also be highly effective against these vectors. In practice, the use of house-derived odours would necessitate at least minor modifications of local houses, such as closing of eave spaces and installation of solar-driven suction fans onto walls or windows, so as to improve efficiency of the suctioning mechanism.

Lastly, even though it was not an objective of this study, it is reasonable to infer from the results that MLB, when fitted with the semi-open screen cage could potentially be used as a monitoring tool for outdoor biting mosquitoes. This approach would, however, require that comparative tests were conducted, which would compare the MLB with current monitoring tools.

## Conclusion

We conclude that while odour-baited devices such as the MLBs clearly have potential against outdoor-biting mosquitoes in communities where LLINs are used, candidate contaminants must be those that are effective at ultra-low doses and on short contacts, since important vector species such as *An. arabiensis* make only brief visits to such devices. Such devices, if coated with suitable mosquito killing agents may complement the existing indoor interventions such as LLINs and IRS by consistently attracting and contaminating or killing vectors outdoors. Natural human odours suctioned from occupied dwellings could constitute affordable sources of attractants to supplement odour baits for the devices. We also recommend that any killing agents used should be environmentally-safe, long-lasting, and have different modes of action (other than pyrethroids as used on LLINs), to mitigate against the risk of physiological insecticide resistance.

## Competing interest

The authors declared that they have no competing interest.

## Authors’ contributions

NSM, JM and FOO conceived the study. NSM, DWL, RDS, EWK, SPM, SM and FOO participated in the design of the experiments. NSM, SM, DRK and EPM performed the experiments. NSM, IM and FOO analysed the data. NSM and FOO wrote the manuscript. All authors read and approved the final manuscript.
